# Chemical Characterization, Antioxidant, Antimicrobial, and Antibiofilm Activities of Essential Oils of *Plumeria alba* (Forget-Me-Not)

**DOI:** 10.1155/2023/1040478

**Published:** 2023-02-22

**Authors:** Kirsty Mary Mawumenyo Mamattah, Abigail Kusiwaa Adomako, Caleb Nketia Mensah, Lawrence Sheringham Borquaye

**Affiliations:** ^1^Department of Chemistry, Kwame Nkrumah University of Science and Technology, Kumasi, Ghana; ^2^Central Laboratory, Kwame Nkrumah University of Science and Technology, Kumasi, Ghana

## Abstract

Essential oils are known to possess many biological properties such as antimicrobial and antioxidant activities. *Plumeria alba* flowers are used in traditional remedies for diarrhea, cough, fever, and asthma treatment. This work evaluated the chemical composition and the biological activities of essential oils obtained from the flowers and leaves of *Plumeria alba*. The essential oils were extracted using the Clevenger-type apparatus and characterized using GC-MS. In the flower essential oil, a total of 17 compounds were identified, with linalool (23.91%), *α*-terpineol (10.97%), geraniol (10.47%), and phenyl ethyl alcohol (8.65%) being abundant. In the leaf essential oil, a total of 24 compounds were identified, with benzofuran, 2,3-di, hydro-(3.24%), and muurolol (1.40%) being present. Antioxidant activities were assessed using hydrogen peroxide scavenging, phosphomolybdenum, and 2, 2-diphenyl-1-picrylhydrazyl (DPPH) free radical-scavenging assays. Antimicrobial activities were assessed through a microdilution assay. The essential oil showed antimicrobial activity against test microorganisms with minimum inhibitory concentrations ranging from 25.0 to 50.0 mg/mL. Biofilm inhibition ranged from 27.14 ± 1.0 to 58.99 ± 0.6 mg/mL. The essential oil exhibited total antioxidant capacities which ranged from 17.5 *μ*g/g AAE to 83 *μ*g/g AAE in the phosphomolybdenum assay. The IC_50_ values in the DPPH and hydrogen peroxide radical scavenging assays for both flowers and leaves ranged from 18.66 *μ*g/mL to 38.28 *μ*g/mL. Both essential oils also displayed good antibiofilm activities, with the concentration required for half-maximal inhibition of biofilm formation being ∼60 mg/mL for both oils. This study shows that essential oils of *Plumeria alba* possess good antioxidant and antimicrobial activities and could be used as a source of natural antioxidants and antimicrobial agents.

## 1. Introduction

Essential oils are predominantly volatile and odorous fractions isolated from plants. These oils usually impart a distinctive and often diagnostic odor to plants. Essential oils have been isolated from various plant parts such as leaves, flowers, and stems (peppermint and lavender), fruits (anise), bark (cinnamon), and seeds (nutmeg). Plants store these oil components in the glandular cells or pockets, which release them with aroma when squeezed or pressed. In plants, essential oils are mostly devoid of cellulose, glycerides, sugars, tannins, salts, and minerals. Conventional methods such as steam distillation, mechanical expression (physical crushing of essential oil glands situated in fruit rinds or outermost waxy layers of fruit's peel), microwave-assisted extraction, solvent extraction, or enfleurage (transfer of the essential oil from flower petals to fat) are usually employed in the extraction of essential oils [1]. Essential oils yields are usually low, often ranging between 0.05 and 18.0% [2]. The yield obtained from the extraction of essential oils depends on various factors, such as the extraction method utilized, environment, climate, soil conditions, time of harvesting, and postharvest handling before isolation [3].

Due to their varied application in food, pharmaceutical, and cosmetics industries, essential oils have become the focus of intense investigations. The pharmaceutical and therapeutic properties of some plants have been attributed to their essential oils and the compounds that make up these oils [4]. In many essential oils, monoterpenes, sesquiterpenes, and aromatic and aliphatic compounds are the principal chemical components. It has been reported that essential oils containing mainly aldehydes or phenols, such as cinnamaldehyde, citral, carvacrol, eugenol, or thymol, are characterized by impressive antibacterial activity [5]. Essential oils with antioxidant, antibiofilm, antitumor, and anti-inflammatory properties have also been reported [6]. The activity of the given essential oil is usually a function of the chemical components present in the oil. Since essential oil composition is affected by cultivation conditions such as soil types and geographical locations and environmental factors like climate and habitat conditions, plants of the same species from different countries may have different chemical compositions and different chemical properties [3]. Due to these, investigations of the essential oils from different plants in different locations have become very important.

The genus *Plumeria* (Apocynaceae) contains a large number of shrubs and flowering trees which are grown throughout the tropics. *Plumeria alba*, locally known as forget-me-not in Ghana, is an essential member of the *Plumeria* genus. It is indigenous to South America and primarily cultivated for its blossoms and fragrant flowers [7]. Ethnomedicinally, different parts of forget-me-not are used in treating several diseases, such as abdominal tumors, leprosy, rheumatism, and malaria. The latex of the leaf and stem is used to treat skin diseases like ulcers, scabies, and herpes [8]. The bark of *P. alba* is used as a plaster for challenging tumors, and the fruit is known as a natural source of antioxidants that can reduce free radical-mediated diseases such as diabetes, cancer, and coronary heart disease [9]. Reports on essential oils from *P. alba* from various countries indicate varying chemical compositions. Lawal et al. in Nigeria identified 43 compounds as the constituents of *P. alba* flower essential oil. Gas chromatography-mass spectrometry (GC/MS) analysis showed that the Nigerian oil was rich in sesquiterpene hydrocarbons (25.2%) with benzyl salicylate (33.98%) as the major constituent [10]. In India, 46 volatile constituents have been characterized in the *P. alba* flower essential oil, accounting for 98.1% of the total essential oil constituents. Esters were the major compound class (48.6%) identified in that study [11]. Other studies have focused on the biological activities of essential oils from *P. alba*. Liu et al. studied the antimicrobial, antioxidant, and antipyretic-analgesic effects of *P. alba* flower essential oil and indicated that the bioactivity may be due to the presence of major constituents such as benzyl salicylate, benzyl benzoate, germacrene B, and linalool in the essential oil [12].

Since essential oils isolated from *P. alba* plant parts in different countries possess varying chemical constituents (qualitative and quantitative) and different biological activities, likely as a result of the differences in chemical composition, the flower and leaf essential oils of Ghanaian cultivars of *P. alba* were studied in this work. Gas chromatography-mass spectrometry was used to identify the essential oils' chemical constituents, whereas standard *in vitro* assays were used to evaluate the antioxidant, antimicrobial, and antibiofilm activities of oils. In the flower and leaf essential oils, 17 and 24 compounds, respectively, were identified, with linalool (flower) and hexadecane (leaf) being the major components. Both oils possessed appreciable antioxidant and antimicrobial activities. Again, both oils were able to inhibit biofilm formation in *Pseudomonas aeruginosa.*

## 2. Methods

### 2.1. Plant Material

Fresh flowers and leaves of *P. alba* were sampled from Kumasi in the Ashanti region of Ghana, in October 2020. The samples were transported to the laboratory at the Department of Chemistry, Kwame Nkrumah University of Science and Technology (KNUST), Kumasi, and stored in a refrigerator at 4°C. Plant identification and authentication were carried out at the Department of Herbal Medicine, KNUST. The specimens of the flowers (KNUST/HMI/2021/F001) and leaves (KNUST/HMI/2021/L011) were deposited at the University Herbarium.

### 2.2. Essential Oil Extraction

Fresh flowers were washed with distilled water. Afterwards, the flowers were subjected to steam distillation for 4 hours in a modified Clevenger-type setup. Fresh leaves were also washed and extracted similarly. The essential oils were recovered and treated with anhydrous sodium sulphate to remove traces of water. Essential oils were stored at 4°C until used in further analysis [13]. The yield of essential oils was calculated with respect to the fresh weight of the plant material before distillation (expressed as the percentage w/w of the fresh material).

### 2.3. Chemical Composition Analysis Using GC-MS

Chemical constituents of the essential oils were assayed on a PerkinElmer GC Clarus 580 gas chromatograph interfaced with a PerkinElmer (Clarus SQ 8 S) mass spectrometer. A DB-5 (ZB-5HTMS; 5% diphenyl/95% dimethylpolysiloxane) fused capillary column with dimensions of 30 × 0.25 mm ID × 0.25 *μ*m DF was used. The GC oven program and the mass spectrometer conditions were the same as reported in previous works [14, 15].

### 2.4. Identification of Compounds

The constituents of the essential oils were identified by matching the mass spectra obtained to mass spectra databases of the National Institute of Standard and Technology (NIST) and Wiley. Where available, mass spectra present in the published literature were also used [16]. Quantitation of essential oil components was by normalization of the peak area of each constituent.

### 2.5. Antioxidant Activity

Various assays were used to evaluate the antioxidant activity of the flower and leaf essential oils of *P. alba.* The assays employed included the inhibition of lipid peroxidation, hydrogen peroxide, and 2,2 diphenyl-1-picrylhydrazyl (DPPH) radical scavenging and phosphomolybdenum assays.

#### 2.5.1. Inhibition of Lipid Peroxidation (Thiobarbituric Acid Reactive Substance, TBARS) Assay

This assay was performed according to methods described by Nartey [15, 17]. The oxidizable substrate used in this assay was the egg yolk, which is known to be rich in lipids. Butylated hydroxytoluene (BHT) was used as the positive control. The percentage inhibition of lipid peroxidation was calculated, and IC_50_ values (extract concentration required to achieve 50% inhibition of lipid peroxidation) were obtained from a graph of % inhibition against concentration.

#### 2.5.2. Phosphomolybdenum Assay

In the phosphomolybdenum (PM) assay, essential oils of different concentrations were prepared in dimethyl sulfoxide (DMSO). Five millimeters of the PM reagent made up of 0.6 M sulfuric acid, 28 mM sodium phosphate, and 4 mM ammonium molybdate was added to 0.5 mL of each test sample in a test tube. The test tube was shaken and then incubated at 95°C for 90 min. After the reaction mixture had cooled to room temperature, the absorbance at 695 nm was measured against a blank solution. Ascorbic acid was used as a standard in this experiment [18, 19].

#### 2.5.3. Hydrogen Peroxide-Scavenging Assay

Effective hydrogen peroxide-scavenging activity of the essential oils was assayed by adding to a test tube 0.5 mL of 1 mM ferrous ammonium sulphate, followed by 0.13 mL of 5 mM H_2_O_2_ and 3 mL of essential oil or a standard drug at varying concentrations. Each test tube was incubated in the dark for 5 min at room temperature. After that, 3 mL of 1 mM 1,10-phenanthroline was added to each mixture, and the test tube was shaken to ensure a uniform mixture. The mixture was then incubated for 10 min at room temperature. Absorbances of the mixtures were taken at 510 nm. Water was used in place of the essential oil in the blank. Ascorbic acid and gallic acid were used as standard drugs. The amount of hydrogen peroxide scavenged was obtained from:

(1) $$\begin{array}{c} \%\;hydr\;ogen\;peroxide\;scavanged=\frac{Atest}{Acontrol}\times 100\text{,} \end{array}$$

where A_test_ is the absorbance of the test sample and A_control_ is the absorbance of the blank.

The IC_50_ values were obtained from a graph of % hydrogen peroxide scavenged against concentration [20].

#### 2.5.4. DPPH Radical-Scavenging Assay

The 2,2-diphenyl-1-picrylhydrazyl (DPPH) free radical-scavenging activities of the flower and leaf essential oils of *P. alba* were evaluated according to a reported method [21, 22]. Methanol was used in place of the essential oils in the negative control set ups. Ascorbic acid was used as a standard drug. The percent inhibition of DPPH free radicals was calculated from the absorbance of the control (Ac) and that of the test (At) from:

(2) $$\begin{array}{c} inhibition\left(\%\right)=\frac{Ac-At}{Ac}\times 100\text{.} \end{array}$$

The IC_50_ values were obtained from a graph of % inhibition against concentration.

### 2.6. Antimicrobial Assay

#### 2.6.1. Microbial Strains

The microbes used were *Staphylococcus aureus* ATCC 29213, *Enterococcus faecalis*, *Bacillus subtilis*, and *Streptococcus pneumoniae* (all Gram-positive bacteria), *Klebsiella pneumoniae* ATCC 700603, *Pseudomonas aeruginosa* ATCC 27853, and *Escherichia coli* TCC 25922 (all Gram-negative bacteria), and *Candida albicans* (a fungus).

#### 2.6.2. Minimum Inhibitory Concentration Determination

The minimum inhibitory concentrations (MIC) of the flower and leaf essential oils were determined using the broth dilution method. In the broth dilution assay, two-fold serial dilution of the essential oil or standard antibiotic (ciprofloxacin) was prepared in sterile 96-well microtiter plates. In each plate, 100 *μ*L of two-fold serial dilution of essential oil was transferred into the wells. To each well was added 100 *μ*L of double-strength nutrient broth containing an inoculum size of ∼2.0 × 10^5^ CFU/mL. After incubation for 24 hours at 37°C, 20 *μ*L of 1.25 mg/mL 3-(4, 5-dimethylthiazol-2-yl)-2,5-diphenyltetrazolium bromide solution (MTT) was added to each well and further incubated for 30 min at 37°C. The MIC was determined as the lowest concentration of the essential oil or antibiotic that completely inhibited the growth of the organism in microdilution wells as detected by the absence of the purple coloration after MTT addition during a 24-hour incubation period at 37°C. All tests were performed in triplicate [23].

### 2.7. Biofilm Inhibition

Biofilm inhibition was investigated in *P. aeruginosa*. A 96-well sterile microtiter plate was filled with 100 *μ*L of various concentrations (MIC and sub-MIC) of essential oils. Gentamicin was used as a standard drug and water as a negative control. Bacterial suspension of *P. aeruginosa* was adjusted to the 0.5 McFarland standard in sterile saline and subsequently inoculated in double-strength nutrient broth to an inoculum size of ∼2.0 × 10 5 CFU/mL. An aliquot of the broth suspension (100 *μ*L) was inoculated into each well to a final volume of 200 *μ*L. The plate was incubated at 37°C for 24 hours. After the incubation period was over, the contents of the plates were emptied and then washed 3 times with deionized water to remove loosely attached cells. The wells were stained with 0.1% (v/v) crystal violet followed by elution with 30% (v/v) glacial acetic acid. This was transferred into a new sterile plate. Absorbance was measured at 595 nm (BioTeK® Synergy H1 multimode microplate reader, Germany). Biofilm inhibition was estimated from equation (2) [24].

### 2.8. Data Analysis

All experiments were conducted in triplicate, and data were presented as a mean ± standard deviation. Statistical analyses were performed in GraphPad Prism 6.0 for Windows. Where applicable, a *p* value <0.05 was considered to be statistically significant.

## 3. Results and Discussion

Essential oils are generally present in trace amounts in plant epidermic cells, secretory cells, canals, and cavities [1]. Varied compounds present in essential oils contribute to the fragrance of the oil. The major compound present in an essential oil may range from 20 to 85% and may contribute greatly to the aroma and pharmacological activity of the essential oil. Compounds present in trace amounts may also act in synergy to elicit specific biological effects [15]. Steam distillation of the flowers and leaves of *P. alba* yielded 0.09% and 0.05% of essential oils, respectively, with both being pale yellow in color. The yield of the flower essential oil is slightly higher than the 0.05% yield as reported by Sahoo et al. in India [11]. An earlier investigation on the leaf and flower essential oil of *P. alba* from Nigeria revealed yields of 0.12% and 0.23%, respectively, with both being significantly greater than the yields obtained in this study [10]. Variations in essential oil yields depend on the method of extraction, age of plant, variety, and plant-growing conditions [3]. These factors may all have played a role in the variations in essential oil yields reported.

**Figure 1:**
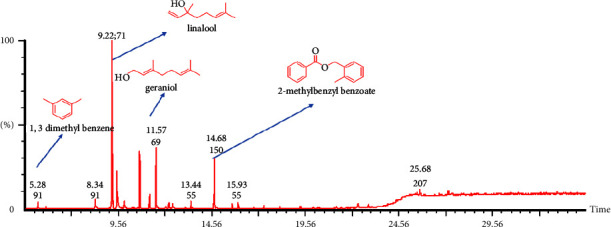
Total ion chromatogram (TIC) obtained from the GC-MS run of the essential oil from the flowers of *Plumeria alba*. Compounds were identified by comparison of MS spectra data with NIST and Wiley libraries.

**Figure 2:**
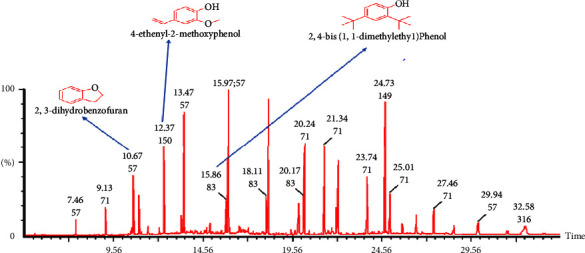
Total ion chromatogram (TIC) obtained from the GC-MS run of the essential oil from the flowers of *Plumeria alba*. Compounds were identified by comparison of MS spectra data with NIST and Wiley libraries.

The total ion chromatograms (TICs) of the flower and leaf essential oils are shown in Figures 1 and 2, respectively, with the composition data of both oils shown in Table 1 (flower essential oil) and Table 2 (leaf essential oil). The compounds present in the flower essential oil were generally primary alcohols, terpenes, terpenoids, ketones, esters, and saturated fatty acids, with terpene and terpenoids dominating. Linalool, *α*-terpineol, and geraniol were the major components present, with their individual compositions being 23.91%, 10.97%, and 10.47% of the total composition, respectively, as shown in Table 1. For the leaf essential oil, 24 compounds were identified and the compounds were mainly carboxylic acids and hydrocarbons. The most abundant component in the leaf essential oils was hexadecane (Table 2). Hexadecane has been found in essential oils from a variety of plants, including *Phlomis lurestanica* [25] and *Cassia Fistula*[26]. In contrast to this study, Lawal identified acyclic monoterpenoids and aldehydes as the main constituents of the leaf oil of *P. alba* in Nigeria [10]. Interestingly, all of the major components of the *P. alba* leaf essential oil reported by Lawal were absent in the leaf essential oil in this study (Table 3). Despite using the same method of extraction, both the yield and composition of the essential oil varied. This variation could be attributed to the difference in geographical locations since essential oil composition is affected by cultivation conditions such as soil types and geographical locations and some environmental factors [3]. Due to these reasons, we suspected that their biological activities may differ since compositions were different.

Essential oils exhibit various biological properties including antioxidant, antimicrobial, and anti-inflammatory. Antioxidants can function by preventing active oxidant generation or scavenging, quenching, and elimination of active oxidants [29]. A formidable challenge in food processing is lipid oxidation. Peroxidation of lipids produces unwanted side products such as aldehydes and peroxides. Oxidized lipid-derived aldehydes are very stable and toxic and have been implicated in a number of human diseases such as carcinogenesis, cardiovascular diseases, neurodegeneration, and aging [30]. In foods, these compounds impart poor sensory properties to the food and contribute significantly to reducing their shelf life. Ultimately, the products of lipid peroxidation enhance food spoilage [31]. Compounds capable of inhibiting lipid peroxidation are therefore desired as they could be used as food preservatives. The TBARS assay was used in assessing lipid peroxidation. This test detects malondialdehyde (MDA), a split product of an endoperoxide of unsaturated fatty acids produced by lipid substrate oxidation [31]. In the lipid peroxidation assay, the IC_50_ value of the flower essential oil was 1943 ± 0.9 *μ*g/mL, while that of the leaf essential oil was 3069 ± 0.9 *μ*g/mL. The standard, BHT, also recorded an IC_50_ value of 9.238 ± 1.7 *μ*g/mL (Table 4). The results imply that the floral essential oil is a better inhibitor of lipid peroxidation than the leaf essential oil. Youdim et al. in 2000 evaluated the antioxidant activity of thyme essential oil and observed that components in the essential oil inhibited lipid peroxidation in the order as follows: p-carvacrol > *γ*-terpinene > myrcene > linalool > p-cymene > limonene > 1,8-cineole > *α*-pinene [32]. Linalool showed a moderate lipid peroxidation inhibition activity and may be a major contributor to the observed inhibition of lipid peroxidation by the flower essential oil of *P. alba* because of its high content in the oil.

The total antioxidant capacity of the essential oil of *P. alba* flower, as determined from the PM assay, was 57 *μ*g/g AAE, whereas that of the leaf essential oil was 87 *μ*g/g AAE. In the hydrogen peroxide-scavenging activity test, the IC_50_ of the flower essential oil was 370.5 *μ*g/mL and that of the *P. alba* leaf essential oil was 476 *μ*g/mL. In the case of the DPPH assay, the concentrations, at which 50% of DPPH radicals were scavenged, were 1014 ± 0.6 and 2798 ± 1.1 *μ*g/mL for the flower and leaf essential oils, respectively (Table 4). The flower essential oil possessed better scavenging activity than the leaf essential oil, whereas the leaf essential oil performed better in the PM assay. Both essential oils could be described as possessing moderate antioxidant activities. Some reports have shown that essential oils possess antioxidant activity, which plays a vital role in neutralizing free radicals and benefiting human health [29, 30, 33]. The antioxidant evaluation of the organic solvent leaf extract of *P. alba* by Siang and et al. revealed moderate antioxidant activity in the DPPH assay, where the IC_50_ was found to be 23.96 mg/mL [34]. Essential oils are therefore better radical scavengers than the organic solvent extracts of the same plant. The antioxidant action of essential oils could be due to the presence of compounds such as linalool, geraniol, and *α*-terpineol in their composition since these compounds are known for their antioxidant properties. Linalool is a principal component in most essential oils with several biological activities which include antioxidant, anti-inflammatory, antibacterial, and antiplasmodial activity [35–37]. The presence of linalool in the flower essential oil as the most abundant component and its complete absence in the leaf essential oil could be a major contributor to the observed patterns in antioxidant capability.

The antimicrobial activity of plant essential oils and extracts has formed the basis for many applications, including raw and processed food preservation, pharmaceuticals, alternative medicine, and natural therapies [6, 38]. Essential oils from various plants have been reported to have impressive antimicrobial properties [6, 14, 39]. Essential oils from the flowers and leaves of *P. alba* were screened against 7 bacteria and 1 fungus. Antimicrobial activity was observed for both Gram-negative and Gram-positive bacteria. However, the essential oils exhibited no antifungal activity at the concentrations used. The results of the antimicrobial susceptibility test (Table 5) show that the MICs of the essential oils against all tested microorganisms ranged between 12.50 and 50 mg/mL. In general, the flower essential oil exhibited better antimicrobial activity when compared to the leaf essential oil. *E. coli* was the most susceptible organism to the flower essential oil at an MIC of 25 mg/mL. The growth of *B. subtilis*, *S. aureus*, *E. faecalis*, and *P. aeruginosa* was also inhibited at 50 mg/mL by the flower essential oil. *E. coli* and *E. faecalis* were susceptible to the leaf essential oil at an MIC of 12.5 mg/mL in both cases. Kumari et al. investigated the antifungal activity of flower essential oils from *P. alba* in India. The results showed that flower essential oil was highly active against *Aspergillus niger*, *Candida albicans*, and *Penicillium chrysogenum* [40]. This was different from what was observed in this study where no activity was observed against *Candida albicans* at the concentrations used. This observation could be attributed to the difference in essential oil composition.

It has been reported that essential oils from the flower of *P. alba* were effective against *S. aureus* and *B. subtilis* [41]. Another report on the activity of the petals of *P. alba* highlighted a significant antimicrobial capacity against pathogenic *E. coli*, one of the most common bacteria with pathogenic strains [42]. In our study, *E. coli* was the most susceptible organism for both essential oils from the flower and leaf of *P. alba*, with the leaf essential oil having a lower MIC than that of the flower essential oil. In general, the flower and leaf essential oils from *P. alba* had a moderate antimicrobial activity against the tested microorganisms. According to several authors, Gram-negative bacteria appear to be less sensitive to the action of many other plant essential oils [43]. This higher resistance among Gram-negative bacteria could be due to the differences in the cell membrane of these bacterial groups. Indeed, the external membrane of Gram-negative bacteria renders their surfaces highly hydrophilic [44], whereas the lipophilic ends of the lipoteichoic acids of the cell membrane of Gram-positive bacteria may facilitate penetration by hydrophobic compounds [45, 46]. Hence, essential oils that can target both bacteria classes will significantly help fight against antimicrobial resistance. Interestingly, in this study, both essential oils of *P. alba* appeared to be active against the tested Gram-positive and Gram-negative bacteria.

Biofilm formation is one of the several strategies utilized by bacteria to evade host immune action and the effects of antimicrobial agents. Biofilm formation has been identified as a plausible strategy used by most bacteria to establish infection in host cells [47]. *P. aeruginosa* is a model biofilm-forming organism that has been well characterized. In this study, *P. aeruginosa* was used for the first time to examine the biofilm inhibition potential of the essential oils from *P. alba*. Because bacterial growth is essential during biofilm development, viable bacteria are needed to develop biofilm in order not to alter biofilm development. For this reason, subinhibitory concentrations of essential oils were used to allow assessment of antimicrobial agents on biofilm formation. Both flower and leaf essential oils displayed good inhibitory effects on biofilm formation, with percent inhibitions ranging from 80 to 41.8% from MIC to sub-MIC doses. At MIC/16, both flower and leaf essential oils inhibited biofilm formation by >40% (Table 6). The concentrations of essential oil, which were determined to affect half-maximal inhibition of biofilm formation (BIC_50_), were 58.99 mg/mL and 60.06 mg/mL, respectively, for flower and leaf essential oils. Thus, the flower essential oil performed better as an antibiofilm agent than the essential oil of the leaf. This was similar to the observation in the antimicrobial studies against *P. aeruginosa*. Extracts of *P. alba* have been shown to possess antibiofilm capabilities. The minimum biofilm inhibitory concentration exhibited by the ethyl acetate extracts of *P. alba* against *P. aeruginosa* was determined to be 60 *μ*g/mL [48]. Thus, both extracts and essential oils from the *P. alba* plant do possess antibiofilm capabilities, although at varying potencies. Several studies have documented the biofilm inhibitory capabilities of essential oils. Essential oils isolated from *Spondias mombin*, *Averrhoa carambola* and *Chrysophyllum albidum* have been reported to possess biofilm inhibition capabilities [14, 15, 17]. Previous studies have also reported the antibiofilm activities of compounds such as linalool, geraniol, and *α*-terpineol [49]. The antibiofilm potential of the *P. alba* essential oils could be due to the presence of some of these compounds.

## 4. Conclusion

The extraction and characterization of essential oils from the flower and leaves of *P. alba* were successful. Terpenoids and terpenes were found in high concentrations in the floral oil, while acids and hydrocarbons were most prevalent in the leaf oil. Both essential oils showed moderate antioxidant and antimicrobial properties, making them potential sources for food preservation and processing, as well as for use in the pharmaceutical and cosmetic industries due to their ability to inhibit biofilm formation.

## Figures and Tables

**Table 1:** Flower essential oil composition of *Plumeria alba* as determined by GC-MS.

S/N	Compound	% composition	RI
1	Linalool	23.91	602
2	Sulfurous acid, dodecyl 2-propyl ester	23.06	2207
3	*α* -Terpineol	10.97	696
4	Geraniol	10.47	758
5	Phenylethyl alcohol	8.65	619
6	Ethanone, 1-(6,6-dimethylbicyclo [3.1.0] hex-2-en-2-yl)-	7.51	991
7	4,5-Dihydro-2 (1H)-pentalenone	2.16	548
8	3,7-Dimethyl-2,6-octadienol	2.08	733
9	Benzene, 1-isocyano-3-methyl-	1.99	645
10	10-Heneicosene	1.96	2385
11	2-Methyl benzyl alcohol	1.64	806
12	m-dimethylbenzene	1.22	203
13	1-Ethyl-2-heptylcyclopropane	1.12	894
14	1-Pentadecanal	0.88	1734
15	2-Butanone, 4-[2-isopropyl-5-methyl-5-(2-methyl-5-oxocyclopentyl) cyclopentenyl]-	0.87	2631
16	3-Tetradecene	0.78	1095
17	3,7,11-Trimethyl-3-hydroxy-6,10-dodecadien-1-yl acetate	0.74	1070

**Table 2:** Leaf essential oil composition of *Plumeria alba* as determined by GC-MS.

S/N	Compound	% composition	RI
1	Hexadecane	11.06	1098
2	Octadecane	9.72	1298
3	Tetradecane	8.32	897
4	Hexacosane	7.85	2105
5	Eicosane	7.15	1450
6	Phytol	6.83	1616
7	Docosane	6.26	1651
8	2-Methoxy-4-vinyl phenol	5.76	816
9	Tetracosane	4.81	1855
10	1,2-Benzenedicarboxylic acid, butyl 8-methyl nonyl ester	4.05	1466
11	Dodecane	4.03	696
12	Benzofuran, 2,3-dihydro-	3.24	717
13	Cetene	2.86	1089
14	E-15-heptadecenal	2.8	1289
15	Hentriacontane	2.72	2613
16	Octacosane	2.16	2426
17	1-Docosene	2.11	1492
18	1,6-Octadien-3-ol, 3,7-dimethyl-	2.06	596
19	Muurolol	1.4	1153
20	1-Hexadecanol	1.33	889
21	Heptadecane	1.02	1195
22	Phenol, 2,4-bis (1,1-dimethyl ethyl)	0.92	1010
23	Decane	0.8	494
24	Octadecanoic acid	0.74	1663

**Table 3:** Comparison of flower and leaf essential oil composition from different studies.

Plant source	Location	Major composition
Flower	Indonesia [27]	Dodecanoic acid (32.51%), tetradecanoic acid (16.96%), hexadecenoic acid (9.11%), dodecanoic acid, ethyl ester (7.56%), octanoic acid (7.28%), and ethanol (6.15%)
	Egypt [28]	Nonadecane (37.69%), 1-nonylcycloheptane (18.39), butylated hydroxytoluene (5.91%), and linoleic acid ethyl ester (5.74%)
	Nigeria [10]	Limonene (9.1%), trans-*α*-bergamotene (8.0%), linalool (7.9%), caryophyllene oxide (7.9%), and (E, E)-*α*-farnesene (6.6)
	India [11]	Benzyl salicylate (33.98%), benzyl benzoate (12.37%), germacrene (10.30%), and linalool (8.71%)
	Present study	Linalool (23.91%), sulfurous acid, dodecyl 2-propyl ester (23.06%), *α*-terpineol (10.97%), geraniol (10.47%), and phenylethyl alcohol (8.65%)

Leaf	Nigeria [10]	Linalool (13.20%), *n*-nonanal (9.60%), phenyl acetaldehyde (8.5%), neryl acetone (5.30%), and *n*-decanal (5.10%)
	Present study	Hexadecane (11.06%), octadecane (9.72%), tetradecane (8.32%), hexacosane (7.85%), eicosane (7.15%), phytol (6.83%), docosane (6.26%), and 2-methoxy-4-vinyl phenol (5.76%)

**Table 4:** Antioxidant activities of flower and leaf essential oils of *Plumeria alba*.

Sample	TAC *μ*g/g AAE	H_2_O_2_ scavenging activity IC_50_ (*μ*g/mL)	DPPH radical-scavenging activity IC_50_ (*μ*g/mL)		TBARS assay IC_50_ (*μ*g/mL)
*P. alba* flowers	57 ± 26.9	370.5 ± 0.5		1014 ± 0.6	1943 ± 0.9
*P. alba* leaves	83 ± 1.41	476.0 ± 0.4		2798 ± 1.1	3069 ± 0.9
Ascorbic acid	ND	5.30 ± 0.2		14.30 ± 4.3	ND
Gallic acid	ND	9.08 ± 1.6		2.26 ± 0.2	ND
BHT	ND	ND		ND	9.238 ± 1.7

Data are represented as a mean ± standard deviation. ^*∗*^TAC: total antioxidant capacity; ^*∗*^TBARS: thiobarbituric acid reactive substance assay; ^*∗*^ND: not determined (compound not used in that experiment).

**Table 5:** Antimicrobial activity and minimum inhibitory concentrations of flower and leaf essential oils of *Plumeria alba* against various microorganisms.

Microorganisms	Flower essential oil (mg/mL)	Leaf essential oil (mg/mL)	Ciprofloxacin (*μ*g/mL)
*Bacillus subtilis* (+)	50	>50	3.125
*Enterococcus faecalis* (+)	50	12.5	3.125
*Staphylococcus aureus* (+)	50	>50	1.563
*Candida albicans*	>50	>50	1.563
*Streptococcus pneumoniae* (−)	>50	>50	3.125
*Klebsiella pneumoniae* (−)	>50	>50	1.563
*Pseudomonas aeruginosa* (−)	50	>50	1.563
*Escherichia coli* (−)	25	12.5	3.125

+, Gram-positive bacteria; −, Gram-negative bacteria.

**Table 6:** Inhibition of *P. aeruginosa* biofilm formation by flower and leaf essential oils of *Plumeria alba*.

Concentrations	% biofilm inhibition		
	Flower essential oil	Leaf essential oil	Gentamicin
MIC	80.21 ± 9.2	64.11 ± 1.9	94.38 ± 6.0
MIC/2	75.76 ± 12.1	58.26 ± 8.3	93.70 ± 6.6
MIC/4	73.47 ± 13.6	55.13 ± 9.1	93.47 ± 6.8
MIC/8	65.08 ± 12.9	54.00 ± 9.1	88.83 ± 1.9
MIC/16	58.40 ± 8.3	52.04 ± 6.9	79.17 ± 6.5
MIC/32	48.46 ± 1.0	41.84 ± 5.9	78.08 ± 6.5
BIC_50_ (mg/mL)	58.99 ± 0.6	60.06 ± 0.9	27.14 ± 1.0

Data are represented as a mean ± standard deviation. Concentrations of essential oils or gentamicin used were based on their minimum inhibitory concentration (MIC) against *P. aeruginosa*. ^*∗*^Gentamicin was in *μ*g/mL doses.

## Data Availability

All data generated or analyzed during this study are included in this published article.
